# Environmental impacts of corn silage production: influence of wheat residues under contrasting tillage management types

**DOI:** 10.1007/s10661-022-10675-8

**Published:** 2022-12-02

**Authors:** Morad Mirzaei, Manouchehr Gorji Anari, Nermina Saronjic, Sudip Sarkar, Iris Kral, Andreas Gronauer, Safwan Mohammed, Andrés Caballero-Calvo

**Affiliations:** 1 Department of Soil Science and Engineering, Faculty of Agricultural Engineering and Technology, University of Tehran, Karaj, Iran; 2 Institute of Soil Research, Department of Forest and Soil Sciences, University of Natural Resources and Life Sciences (BOKU), Vienna, Austria; 3 ICAR Research Complex for Eastern Region, Patna, 800014 India; 4 Institute of Agricultural Engineering, Department of Sustainable Agricultural Systems, University of Natural Resources and Life Sciences, Vienna, Austria; 5 Institute of Land Utilization, Technology and Regional Planning, Faculty of Agricultural and Food Sciences and Environmental Management, University of Debrecen, Debrecen, Hungary; 6 Departamento de Análisis Geográfico Regional y Geografía Física, Facultad de Filosofía y Letras, Universidad de Granada, Campus Universitario de Cartuja, 18071 Granada, Spain

**Keywords:** Sustainable agriculture, Crop residues, Life cycle assessment, Soil management

## Abstract

The intensification of specific land management operations (tillage, herbicide, etc.) is increasing land degradation and contributing to ecosystem pollution. Mulches can be a sustainable tool to counter these processes. This is particularly relevant for rural areas in low-income countries where agriculture is a vital sector. In this research, the environmental impact of different rates of wheat residues (no residues, 25, 50, 75, and 100%) in corn silage cultivation was evaluated using the life cycle assessment (LCA) method under conventional tillage (CT) and no-tillage (NT) systems in a semi-arid region in Karaj, Iran. Results showed that in both tillage systems, marine aquatic ecotoxicity (ME) and global warming potential (GWP) had the highest levels of pollution among the environmental impact indicators. In CT systems, the minimum (17,730.70 kg 1,4-dichlorobenzene (DB) eq.) and maximum (33,683.97 kg 1,4-DB eq.) amounts of ME were related to 0 and 100% wheat residue rates, respectively. Also, in the CT system, 0 and 100% wheat residue rates resulted in minimum (176.72 kg CO_2_ eq.) and maximum (324.95 kg CO_2_ eq.) amounts of GWP, respectively. However, in the NT system, the 100% wheat residue rate showed the minimum amounts of ME (11,442.39 kg 1,4-DB eq.) and GWP (120.21 kg CO_2_ eq.). Also, in the NT system, maximum amounts of ME (17,174 kg 1,4-DB eq.) and GWP (175.60 kg CO_2_ eq.) were observed with a zero wheat residue rate. On-farm emissions and nitrogen fertilizers were the two factors with the highest contribution to the degradation related to environmental parameters at all rates of wheat residues. Moreover, in the CT system, the number of environmental pollutants increased with the addition of a higher wheat residue rate, while in the NT system, increasing residue rates decreased the amount of environmental pollutants. In conclusion, this LCA demonstrates that the NT system with the full retention of wheat residues (100%) is a more environmentally sustainable practice for corn silage production. Therefore, it may be considered one of the most adequate management strategies in this region and similar semi-arid conditions. Further long-term research and considering more environmental impact categories are required to assess the real potential of crop residues and tillage management for sustainable corn silage production.

**Supplementary Information:**

The online version contains supplementary material available at 10.1007/s10661-022-10675-8.

## Introduction

Sustainable intensification of crop production is crucial for increasing global demand for food production with minimum environmental impact (Ball et al., 2005; Garnett et al., 2013; Struik & Kuyper, 2017; Xie et al., 2019). Recently, it has been demonstrated that silage crops can contribute to the achievement of production targets such as growth or weight gain of livestock and may compensate for seasonal shortfalls between feed demand and supply (Crotty et al., 2016). They can also play an important role in maintaining ground cover, preventing erosion, accumulating nitrogen in the soil, and improving the diversity and abundance of soil biota and the soil condition (Crotty et al., 2015, 2016; van Eekeren et al., 2009). Corn silage is a plant material (mainly plant leaves and stems) used in stockbreeding. In Iran, the cultivation area of silage crops was about 280,000 ha with an average yield of 70 t ha^−1^ (Ministry of Jihad-e-Agriculture of Iran, 2018). Nowadays, this country is being affected by numerous land degradation processes in cultivated areas such as erosion, soil pollution, and loss of biodiversity due to non-sustainable land management practices such as intensive tillage operations, removal and burning of crop residues, monocropping, excessive use of chemical fertilizers, and lack of using green manures/cover crops (Sadeghi et al., 2008; Nawaz et al., 2017; Minaei et al., 2018; Javidan et al., 2019; Mirzaei et al., 2021; Mohammed et al., 2022; Eskandari Dameneh et al., 2021; Rodrigo-Comino et al., 2022).

Exacerbated intensification and non-planned farming practices contribute significantly to increasing soil consumption and gas emissions to the environment (Mirzaei et al., 2022a, b; Rodrigo-Comino et al., 2020; Smith et al., 2017, 2021). However, to achieve land degradation neutrality in these areas considering the effects on carbon and nitrogen dynamics, agricultural practices such as reduced tillage and the use of crop residues could have direct (i.e., carbon sequestration, mitigation of greenhouse gases emissions, absorbing pollutants and other chemicals used in agriculture, reducing air pollutants) and indirect (i.e., reducing fuel and energy consumption) positive effects on global warming and environmental quality (Brennan et al., 2014; Liu et al., 2014; Mirzaei et al., 2022a; Ravindra et al., 2019; Sindelar et al., 2019; Yao et al., 2013).

In general, crop residues are parts of the plants that remain in the field after harvest. Around 3.5–4 × 10^9^ megagrams (Mg) of plant residues are generated every year around the world. Approximately 75% of this amount can be attributed to cereals (Bhattacharyya & Barman, 2018). Crop residues are removed from the field after harvest in many parts of the world (Cherubin et al., 2018; Kenney et al., 2015; Mirzaei et al., 2021; Turmel et al., 2015; Wegner et al., 2015; Wilhelm et al., 2004). The removed residues are generally used for food and fiber (animal feed and bedding, biofuel production, building materials, household fuel, paper making, and mushroom cultivation), which adversely affect soil fertility, agronomic productivity, and environmental quality (Maw et al., 2019; Mirzaei et al., 2021). Moreover, post-harvest crop residue burning is performed by farmers in many parts of the world with serious health and environmental consequences (Chen et al., 2019; Mousavi-Avval et al., 2017a).

In Iran, one of the most widely performed cropping practices is the wheat–corn rotation system. Annually, a massive amount of crop residues is produced but, unfortunately, these residues are burned or removed for fodder, energy production, or other purposes (Mirzaei et al., 2021; Rasoulzadeh et al., 2019). Furthermore, extensive use of conventional tillage systems, chemical fertilizers, and lack of proper crop rotation have caused a decline in organic matter, poor soil quality, lower crop performance, emission of greenhouse gases, and environmental pollution in the region (Mirzaei et al., 2022a, b; Naseri et al., 2021). However, the implementation of conservation tillage methods and adequate management of crop residues can improve soil quality and crop production, as well as contribute to the mitigation of environmental impacts (Cerdà et al., 2016; Nunes et al., 2016; Rakkar et al., 2017; Whitbread et al., 2000). Therefore, an evaluation of the environmental sustainability of different tillage methods and crop residue management practices in specific cropping systems is necessary.

In recent years, life cycle assessment (LCA) has been applied as a standard method to assess the environmental impacts and to analyze the sustainability of the production systems from the environmental point of view (Iriarte et al., 2010; Mousavi-Avval et al., 2017b). In different parts of the world, the LCA of agricultural products has been conducted. Reviewing the literature shows that there are some studies on the LCA approach. Table S1 shows some of these studies in crop production.

Appropriate management of crop residues in cropping systems and their associated high environmental impacts is important in Iran. Therefore, detailed analyses of environmental parameters are necessary to achieve decision-making goals in agriculture. For this purpose, the main goal of this research is to assess environmental indicators for corn silage production in terms of varying wheat residue rates under conventional and no-tillage systems using the LCA approach. To achieve a supportive sustainable phase, the main goals of this research are: as follows.

i. Decomposition and assessment of LCA analysis in corn silage production
ii. Selection of the hotspot from inputs to increase environmental impacts in the application of corn silage production
iii. Comparison of environmental impact categories attained with LCA, calculated in both conventional and no-tillage systems for corn silage production under different rates of wheat residues, and
iv. The selection of more sustainable residue rates considering each tillage system

## Methodology

### Site characteristics and experimental design

The research was conducted in 2018 at the Agriculture Research Station of the College of Agriculture and Natural Resources, University of Tehran, Karaj, Iran (35° 48ʹ 32″ N, 50° 58ʹ 06″ E, 1308 m a.s.l., mean average temperature of 13.7 °C, and mean average precipitation of 245.5 mm). Two fields with two different management systems, i.e., conventional tillage (CT) and no-tillage (NT), have been used for the study. The properties of the soil in both tillage systems before the start of the experiment are presented in Table S2. The NT system was applied 7 years before the start of the research. Both fields had a minimum of 15 years under the corn (*Zea mays* L.)–wheat (*Triticum aestivum* L.) rotation system. For each of the tillage management systems, the field size was 19 × 22 m. Each field was divided into 20 plots of 3 m × 4 m. The research model was based on a randomized complete block with four replications for each treatment.

### Soil treatments, implementation, planting operations, and data collection

Wheat residue treatments were applied in both the NT and the CT fields following the wheat harvest from the previous year. Five different rates of wheat residues (Table S3) were applied, i.e., 100% (3.5 t ha^−1^), 75% (2.625 t ha^−1^), 50% (1.75 t ha^−1^), 25% (0.875 t ha^−1^), and no residue. The selection of wheat residue rates was based on the weight of post-harvest crop residues left on the farm. To determine the weight of post-harvest wheat residues, a wooden quadrat (1 m × 1 m) was used. The post-harvest wheat residues were sampled at several points in each farm, and the average amount of these points was considered as the amount of residue per m^2^ and, finally, scaled up to a 1-ha basis. In both NT and CT fields, the wheat residues were distributed uniformly over the surface of the plots. Then, corn (*Zea mays* L.), a cultivar of Single Cross 704, was sown in July 2018 using a row-crop planter with 75-cm spacing between the rows. In the no-tillage field, seed placement was carried out using a planter with a single colter to cut through residues and loosen the soil before the standard planter placed seeds. However, in the conventional tillage field, the soil has been cultivated to a depth of 35 cm by moldboard plowing, followed by disk harrowing and land leveling which were performed to break up clods and level the land, respectively. Then, seed placement was done using a row-crop planter. Basal NPK fertilizers were spread in the form of 50 kg urea, 70 kg potassium sulfate, and 150 kg superphosphate per ha. Additional N was top dressed at eight-leaf (80 kg urea ha^−1^) and ten-leaf (270 kg urea ha^−1^) stages. The fields were irrigated after planting and on a 7–10-day interval. Irrigation was carried out using sprinkler and furrow systems in NT and CT systems, respectively. Weeds were removed manually at four-leaf and eight-leaf stages. The climatic data during the growing season are presented in Table S4. Required information on silage corn production during the experimental period was recorded in both CT and NT systems. Input data included human labor, machinery, diesel, chemical fertilizers, electricity, water, wheat residues, and seed.

### Soil and wheat residue analyses

Before the start of the experiment in July 2018, soil samples were collected from several points in each CT and NT system for 0–10 and 10–20 cm soil depths. For each soil depth, the soil samples were uniformly mixed to make one composite sample and subsequently were air-dried, sieved (2 mm), and analyzed. Soil pH and electrical conductivity (EC) were measured in saturated soil extracts, and Walkley and Black (1934) method was used to determine soil organic carbon (SOC). Available potassium and phosphorus were determined using ammonium acetate and NaHCO_3_ methods, respectively (Knudsen et al., 1982; Page et al., 1982). Total nitrogen (TN) was measured using the method described by Bremner and Mulvaney (1965). Soil bulk density was measured using stainless steel cylinders (100 cm^3^ volume). Soil texture was measured by the hydrometric method (Gee & Bauder, 1986).

The wheat residues were analyzed for their elemental composition. For this purpose, the residues were dried and finely powdered for analysis. Organic carbon (OC) was determined using the Walkley and Black method (1934). The potassium (K) and phosphorus (P) contents in the residues were measured on dry-ashed samples using spectrophotometric and flame photometric methods, respectively (Jones, 2001). The Kjeldahl method was used to determine the TN in wheat residues (Jones, 2001).

### Yield determination

Corn silage yield was determined in October 2018. After excluding the plot margins, a 1 m × 1 m wooden quadrat was used to harvest three locations within each plot. To calculate corn silage yield, the whole plants were harvested, numbered, and weighed for each plot separately. Their mean was calculated as corn silage yield per m^2^ and, finally, scaled up to a 1-ha basis.

### LCA approach

LCA is a standard approach for evaluating environmental aspects and analyzing the sustainability of production systems (Mousavi-Avval et al., 2017b). LCA is an analytical method usually concentrated on resource consumption and impacts on human health, as well as on the environment associated with the manufacturing of particular products and services (Ghasemi-Mobtaker et al., 2020). In agricultural systems, the main steps of LCA are (1) definition of scope (determining system boundaries, and parameters), (2) inventory analysis (identifying inputs and outputs of all processes in the life cycle), (3) impact assessment (setting assessment criteria; quantifying the environmental impact), and (4) data interpretation (analyzing and comparing all impacts and performing sensitivity analysis) (Kaab et al., 2019b; Recanati et al., 2018).

#### Goal and scope definition

**Fig. 1:**
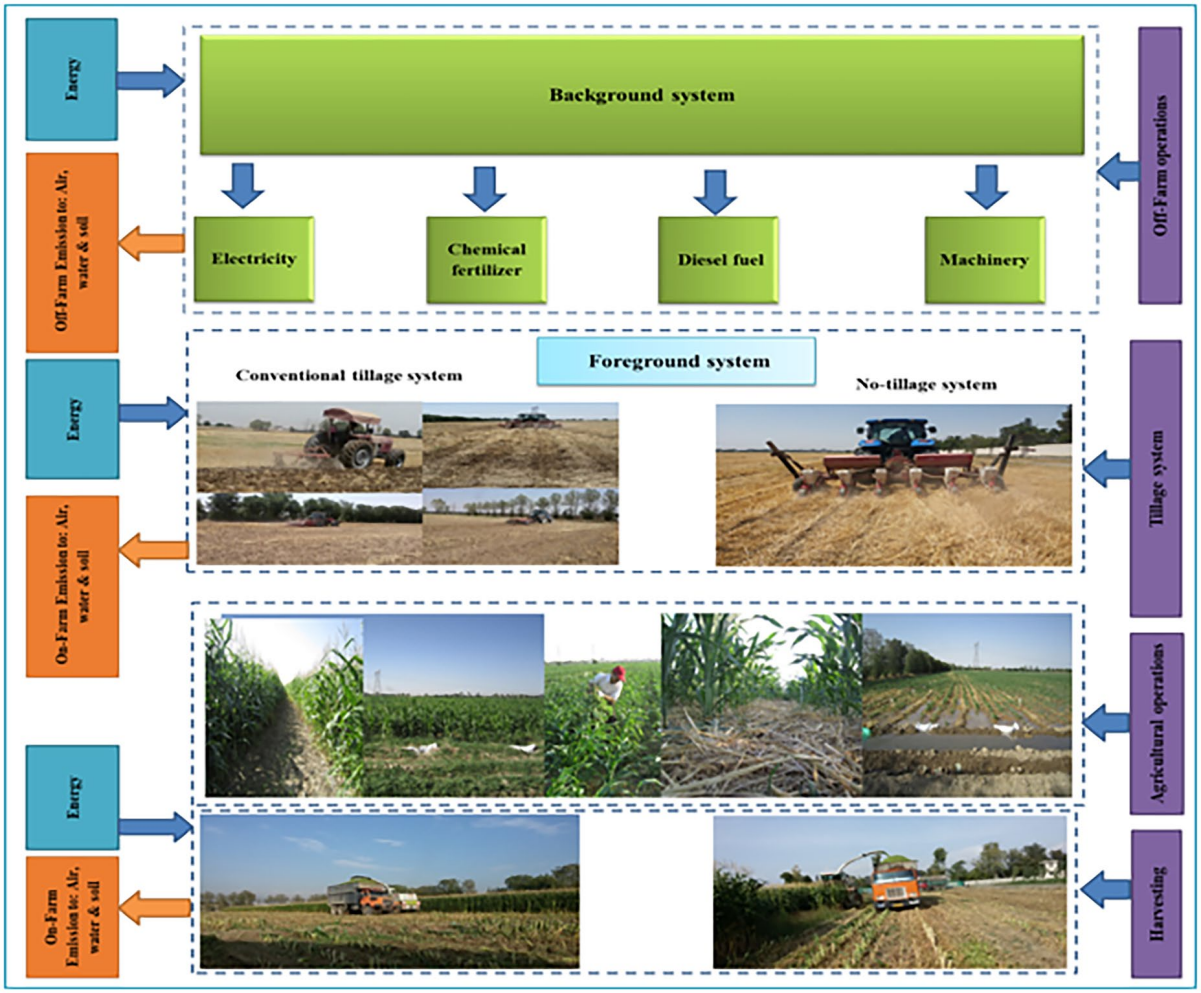
System boundaries of corn silage production under CT and NT systems

At the stage of defining the purpose and scope of an ISO standard LCA, the aim of the evaluation is determined and decisions are made about the details of the studied product system. Before the collection of any data, the goal and scope are defined at the outset of the study (Curran, 2017). All environmental impacts were calculated for the production of 1 ton of corn silage as FU (see the list of abbreviations). The study boundaries considered for different corn silage cultivation systems in the present study are defined in Fig. 1.

#### Life cycle inventory

Life cycle inventory (LCI) is defined as the quantification of the inputs and outputs of a system including its material and energy flows. LCI is divided into two categories, including off-farm and on-farm emissions (ISO, 2006).

##### Off-farm emissions

In this research, the uses of electricity, seeds, chemical fertilizers, diesel, machinery, and wheat residues are considered the inputs of the system. The output includes corn silage. The inputs as indirect emissions were used in the life cycle of corn silage production.

##### On-farm emissions

Emissions to the air in corn silage production are mostly caused by agricultural machinery such as tractors and tillers used for farm practices such as fertilization and plowing. Traction was used to estimate the emissions that originated from the use of machinery and emissions of diesel combustion. Traction is calculated in megajoules (MJ) and contains all diesel consumption. In this study, the amount of different emission factors derived from data, as shown in Table 1 (Nabavi-Pelesaraei et al., 2017a).

**Table 1:** The equivalent of direct emission of 1 megajoule (MJ) diesel for 1 MJ burning in the Ecoinvent database

Emission	Amount (g MJ^−1^ diesel)
CO_2_	74.5
SO_2_	2.41E − 02
CH_4_	3.08E − 03
Benzene	1.74E − 04
Cd	2.39E − 07
Cr	1.19E − 06
Cu	4.06E − 05
N_2_O	2.86E − 03
Ni	1.67E − 06
Zn	2.39E − 05
Benzo[a]pyrene	7.16E − 07
NH_3_	4.77E − 04
Se	2.39E − 07
PAH	7.85E − 05
HC, as NMVOC	6.80E − 02
NO_*x*_	1.06
CO	1.50E − 01
Particulates (b2.5 μm)	1.07E − 01

The emissions to the air and water in corn silage production are related to the chemical fertilizers consumed and human labor used. To calculate these emissions, input amounts were multiplied by their emission factors. The equivalent coefficients of the inputs are shown in Table 2.

**Table 2:** Coefficients for calculating the direct emissions to the soil of heavy metals related to the application of chemical fertilizers in corn silage production (Durlinger et al., 2015)

Characteristic		Heavy metals						
		Cd	Cu	Zn	Pb	Ni	Cr	Hg
1	$\frac{{\text{mg Heavy metal}}}{{{\text{kg N}}_{{\text{in fertilizer applied}}} }}$	6	26	203	5409	20.9	77.9	0.1
2	$\frac{{\text{mg Heavy metal}}}{{{\text{kg P}}_{{\text{in fertilizer applied}}} }}$	90.5	207	1923	154	202	1245	0.7
3	$\frac{{\text{mg Heavy metal}}}{{{\text{kg K}}_{{\text{in fertilizer applied}}} }}$	0.2	8.7	11.3	1.5	4.5	10.5	0.1

Emissions from the soil in corn silage production are related to the application of chemical fertilizers, which release heavy metals into the soil. These types of direct emissions were calculated by multiplying the amount of the inputs for chemical fertilizers by their emission factors. The equivalent coefficients are shown in Table 3.

**Table 3:** Coefficients for calculating the direct emissions related to the application of inputs in corn silage production

Characteristic		Coefficient (emission result)	Reference
*A. Emissions of fertilizers*			
1	$\frac{{{\text{kg N}}_{{2}} {\text{O - N}}}}{{{\text{kg N}}_{{\text{in fertilizers applied}}} }}$	0.01 (to air)	IPCC (2006)
2	$\frac{{{\text{kg NH}}_{{3}} {\text{ - N}}}}{{{\text{kg N}}_{{\text{in fertilizers applied}}} }}$	0.1 (to air)	IPCC (2006)
3	$\frac{{{\text{kg N}}_{{2}} {\text{O - N}}}}{{{\text{kg N}}_{{\text{in atmospheric deposition}}} }}$	0.001 (to air)	IPCC (2006)
4	$\frac{{{\text{kg NO}}_{{3}}^{ - } {\text{ - N}}}}{{{\text{kg N}}_{{\text{in fertilizers applied}}} }}$	0.1 (to water)	IPCC (2006)
5	$\frac{{\text{kg P emission}}}{{{\text{kg P}}_{{\text{in fertilizers applied}}} }}$	0.02 (to water)	IPCC (2006)
6	$\frac{{{\text{kg NO}}_{x} }}{{{\text{kg N}}_{{2}} {\text{O}}_{{\text{from fertilizers and soil}}} }}$	0.21 (to air)	IPCC (2006)
*B. Conversion of emissions*			
1	Conversion from kg CO_2_-C to kg CO_2_	$\frac{44}{{12}}$	IPCC (2006)
2	Conversion from kg N_2_O-N to kg N_2_O	$\frac{44}{{28}}$	IPCC (2006)
3	Conversion from kg NH_3_-N to kg NH_3_	$\frac{17}{{14}}$	IPCC (2006)
4	Conversion from kg NO_3_-N to kg NO_3_	$\frac{62}{{14}}$	IPCC (2006)
5	Conversion from kg P_2_O_5_ to kg P	$\frac{62}{{164}}$	IPCC (2006)
*C. Emissions from human labor*			
1	$\frac{{{\text{kg CO}}_{{2}} }}{{{\text{man - human labor (h}})}}$	0.7 (to air)	Mousavi-Avval et al. (2017a)

### Life cycle impact assessment

Life cycle impact assessment (LCIA) is a method used to elucidate the severity of LCI outcomes in relation to their environmental impacts, such as climate change or human health (Yin, 2019).

In this study, the CML 2 baseline was applied. Another purpose of the assessment was the interpretation of the inputs and outputs of the corn silage production system.

#### The CML 2 baseline method

The CML 2 baseline is an impact assessment method that restricts quantitative modeling to the early stages of the cause–effect chain to limit uncertainties (Khanali et al., 2016). The CML 2 baseline method offers the option of analyzing the environmental burden under ten impact category areas, including abiotic depletion (AD), acidification (AC), eutrophication (EP), global warming potential (GWP), ozone layer depletion (OLD), human toxicity (HT), freshwater aquatic ecotoxicity (FE), marine aquatic ecotoxicity (ME), terrestrial ecotoxicity (TE), and photochemical oxidation (PO) (Kaab et al., 2019a, b; Milutinović et al., 2017; Romero-Gámez et al., 2014). Previous studies have also used these impact categories frequently (Ghasemi-Mobtaker et al., 2020; Kaab et al., 2019a; Kouchaki-Penchah et al., 2016; Nabavi-Pelesaraei et al., 2019). For the calculation of these impact categories, the data of LCI (Table 4) was introduced into the SimaPro software (Kaab et al., 2019a). SimaPro software is one of the widely used applications for LCA (Abeliotis et al., 2013).

**Table 4:** LCI of CT and NT systems by different rates of wheat residue in corn silage production, based on 1 ha

Item (unit)	CT					NT				
	Wheat residue					Wheat residue				
	0%	25%	50%	75%	100%	0%	25%	50%	75%	100%
*A. Off-farm emission*										
1. Human labor (h)	100	100	100	100	100	40	40	40	40	40
2. Machinery (kg)	120	120	120	120	120	80	80	80	80	80
3. Diesel (kg)	170	170	170	170	170	60	60	60	60	60
4. Chemical fertilizers (kg)										
(a) Nitrogen (N)	185	185	185	185	185	185	185	185	185	185
(b) Phosphate (P_2_O_4_)	30	30	30	30	30	30	30	30	30	30
(c) Potassium (K)	30	30	30	30	30	30	30	30	30	30
5. Electricity (kWh)	1525	1525	1525	1525	1525	530	530	530	530	530
6. Water (m^3^)	7000	7000	7000	7000	7000	4800	4800	4800	4800	4800
7. Wheat residue (tons/ha)	0	0.875	1.75	2.625	3.5	0	0.875	1.75	2.625	3.5
8. Seed (kg)	35	35	35	35	35	35	35	35	35	35
*B. On-farm emission*										
1. Emissions by diesel to air (kg)										
(a) CO_2_	613.32	613.32	613.32	613.32	613.32	216.46	216.46	216.46	216.46	216.46
(b) SO_2_	0.19	0.19	0.19	0.19	0.19	0.07	0.07	0.07	0.07	0.07
(c) CH_4_	0.02	0.02	0.02	0.02	0.02	0.0089	0.0089	0.0089	0.0089	0.0089
(d) Benzene	0.001	0.001	0.001	0.001	0.001	0.0005	0.0005	0.0005	0.0005	0.0005
(e) Cd	1.96E − 06	1.96E − 06	1.96E − 06	1.96E − 06	1.96E − 06	6.94E − 07	6.94E − 07	6.94E − 07	6.94E − 07	6.94E − 07
(f) Cr	9.7E − 06	9.7E − 06	9.7E − 06	9.7E − 06	9.7E − 06	3.45E − 06	3.45E − 06	3.45E − 06	3.45E − 06	3.45E − 06
(g) Cu	0.0003	0.0003	0.0003	0.0003	0.0003	0.0001	0.0001	0.0001	0.0001	0.0001
(h) N_2_O	0.02	0.02	0.02	0.02	0.02	0.0083	0.0083	0.0083	0.0083	0.0083
(i) Ni	1.37E − 05	1.37E − 05	1.37E − 05	1.37E − 05	1.37E − 05	4.8E − 06	4.8E − 06	4.8E − 06	4.8E − 06	4.8E − 06
(j) Zn	0.00019	0.00019	0.00019	0.00019	0.00019	6.9E − 05	6.9E − 05	6.9E − 05	6.9E − 05	6.9E − 05
(k) Benzo[a]pyrene	5.8E − 06	5.8E − 06	5.8E − 06	5.8E − 06	5.8E − 06	2.08E − 06	2.08E − 06	2.08E − 06	2.08E − 06	2.08E − 06
(l) NH_3_	0.003	0.003	0.003	0.003	0.003	0.0013	0.0013	0.0013	0.0013	0.0013
(m) Se	1.96E − 06	1.96E − 06	1.96E − 06	1.96E − 06	1.96E − 06	6.94E − 07	6.94E − 07	6.94E − 07	6.94E − 07	6.94E − 07
(n) PAH	0.00064	0.00064	0.00064	0.00064	0.00064	0.0002	0.0002	0.0002	0.0002	0.0002
(o) HC, as NMVOC	0.55	0.55	0.55	0.55	0.55	0.19	0.19	0.19	0.19	0.19
(p) NO_*x*_	8.72	8.72	8.72	8.72	8.72	3.07	3.07	3.07	3.07	3.07
(q) CO	1.23	1.23	1.23	1.23	1.23	0.43	0.43	0.43	0.43	0.43
(r) Particulates (b2.5 μm)	0.88	0.88	0.88	0.88	0.88	0.31	0.31	0.31	0.31	0.31
2. Emissions by fertilizers to air (kg)										
(a) N_2_O	2.91	3.10	3.14	3.38	3.57	2.91	3.02	3.13	3.24	3.35
(b) NH_3_	22.55	24.01	24.33	26.12	27.59	22.55	23.39	24.23	25.07	25.91
3. Emission by atmospheric deposition of fertilizers to air (kg)										
(a) N_2_O	0.28	0.30	0.30	0.32	0.34	0.28	0.29	0.31	0.32	0.33
4. Emissions by fertilizers to water (kg)										
(a) Nitrate	24.67	26.27	26.62	28.58	30.19	24.67	25.59	26.51	27.43	28.35
(b) Phosphate	0.40	0.40	0.40	0.40	0.40	0.40	0.40	0.40	0.40	0.40
5. Emission of nitrogen in incorporating wheat residue with soil to air (kg)										
(a) N_2_O	0	0.11	0.23	0.34	0.462	0	0.05	0.11	0.17	0.23
6. Emission by N_2_O of fertilizers and soil to air (kg)										
(a) NO_*x*_	39.00	41.53	42.08	45.17	47.72	39	40.45	41.91	43.36	44.81
7. Emission by human labor to air (kg)										
(a) CO_2_	70	70	70	70	70	28	28	28	28	28
8. Emission by heavy metals of fertilizers to soil (g)										
(a) Cd	15.989	16.16968	16.35032	16.53104	16.71174	15.989	16.07934	16.16968	16.26002	16.35037
(b) Cu	42.059	42.84196	43.62472	44.40784	45.19087	42.059	42.45048	42.84196	43.23342	43.62493
(c) Zn	370.441	376.55414	382.66566	388.78002	394.89367	370.441	373.49757	376.55414	379.61051	382.66728
(d) Pb	2186.805	2349.69163	2512.53498	2675.45406	2838.35421	2186.805	2268.24831	2349.69163	2431.12953	2512.57825
(e) Ni	38.975	39.60438	40.23360	40.86311	41.49254	38.975	39.28969	39.60438	39.91905	40.23377
(f) Cr	218.645	220.99088	223.33614	225.68249	228.02856	218.645	219.81794	220.99088	222.16374	223.33676
(g) Hg	0.152	0.15501	0.15802	0.16103	0.16405	0.152	0.15351	0.15501	0.15652	0.15802
*C. Forage corn yield (fresh weight, kg)*	50,850	51,725	52,875	53,325	54,050	56,925	57,038	60,978	61,088	61,550

### Interpretation of results

Life cycle interpretation is a systematic part of the LCA method to identify, quantify, check, and assess the information derived from the results of the LCI and/or the LCIA. The results of the inventory analysis and impact evaluation are presented in the interpretation phase (Cao, 2017). The outcome of the interpretation phase is a set of conclusions and recommendations for the study. Three main options should be included for the interpretation according to ISO (ISO, 2006): (1) identification of important issues based on the results of the LCI and LCIA stages of an LCA; (2) assessment of the study by taking into account completeness, sensitivity, and compatibility studies; and (3) conclusions, limitations, and recommendations. In this study, LCA analyses are conducted using an Excel 2016 spreadsheet and SimaPro software.

LCI analysis involves creating an inventory of flows from the nature of a product system. Inventory flows include inputs of water, energy, and raw materials, which are released into the environment. In this study, the LCI is divided into two main sections, including inputs and outputs of corn silage cultivation under CT and NT systems for different rates of wheat residues and also off-farm and on-farm emissions.

## Results

### LCI analysis

The amounts of different inputs (including human labor, machinery, diesel, chemical fertilizer, electricity, water, wheat residues, and seed required for producing corn silage per hectare) that were used in the LCA analysis are shown in Table 4. The amount of inputs for the CT system is higher than that for the NT system. Agricultural practices, such as the addition or removal of wheat residues and land preparation after wheat harvesting, are time-consuming and, hence, require more working hours for machinery, resulting in higher fuel consumption. Table 4 also shows on-farm emissions in the process of corn silage production per hectare under different systems and different rates of wheat residues. The highest amount of CO_2_ released from diesel is associated with the CT system due to the high consumption of diesel from land preparation for harvest. An increase in the wheat residues will also increase the nitrate emissions from nitrogen consumption. Also, the highest levels of emissions by heavy metals from chemical fertilizers were associated with the CT system. The product yield in both systems increases with raising wheat residues. The amount of corn silage yield in the NT system was higher than that in the CT system. These results indicate that the use of wheat residues on the soil surface leads to an increase in soil organic matter, water use efficiency, and yield of crops under this study.

### Environmental impact results

#### LCA analysis of corn silage in the CT system

The effects of wheat residue rates on different environmental impact categories under the CT system in corn silage production are displayed in Table 5. Results showed that the raising of wheat residues led to an increase in the number of environmental pollutants. ME and GWP have the highest levels of pollution among all the studied environmental impacts. The amount of ME in different rates of wheat residues, i.e., 0, 25, 50, 75, and 100% wheat residues, was calculated as 17,730.7 kg, 20,167.34 kg, 21,263.32 kg, 27,497.23 kg, and 33,683.97 kg 1,4-dichlorobenzene (DB) eq., respectively. Furthermore, the amounts of GWP were calculated as 176.72 kg, 199.18 kg, 205.87 kg, 267.68 kg, and 324.95 kg CO_2_ eq., respectively, in identical wheat residues. The highest environmental impacts are related to the circumstance comprising 100% rate of wheat residues.

**Table 5:** Results for the environmental impacts of the CT system for different rates of wheat residue to produce 1 ton of corn silage

Impact categories	Measurement units	Value				
		0% wheat residue	25%	50%	75%	100%
AD	kg Sb eq	0.66	0.75	0.80	1.03	1.27
AC	kg SO_2_ eq	2.02	2.22	2.25	2.89	3.42
EP	kg ${\text{PO}}_{4}^{3 - }$ eq	0.48	0.53	0.53	0.68	0.80
GWP	kg CO_2_ eq	176.72	199.18	205.87	267.68	324.95
OLD	kg CFC-11 eq	4.22E − 06	4.81E − 06	5.08E − 06	6.57E − 06	8.06E − 06
HT	kg 1,4-DB eq	91.39	102.99	107.54	137.82	167.20
FE	kg 1,4-DB eq	15.86	17.99	18.93	24.43	29.85
ME	kg 1,4-DB eq	17,730.70	20,167.34	21,263.32	27,497.23	33,683.97
TE	kg 1,4-DB eq	3.76	4.16	4.26	5.36	6.37
PO	kg C_2_H_4_ eq	0.03	0.03	0.04	0.05	0.06

*AD* abiotic depletion, *AC* acidification, *EP* eutrophication, *GWP* global warming potential, *OLD* ozone layer depletion, *HT* human toxicity, *FE* freshwater aquatic ecotoxicity, *ME* marine aquatic ecotoxicity, *TE* terrestrial ecotoxicity, *PO* photochemical oxidation

**Fig. 2:**
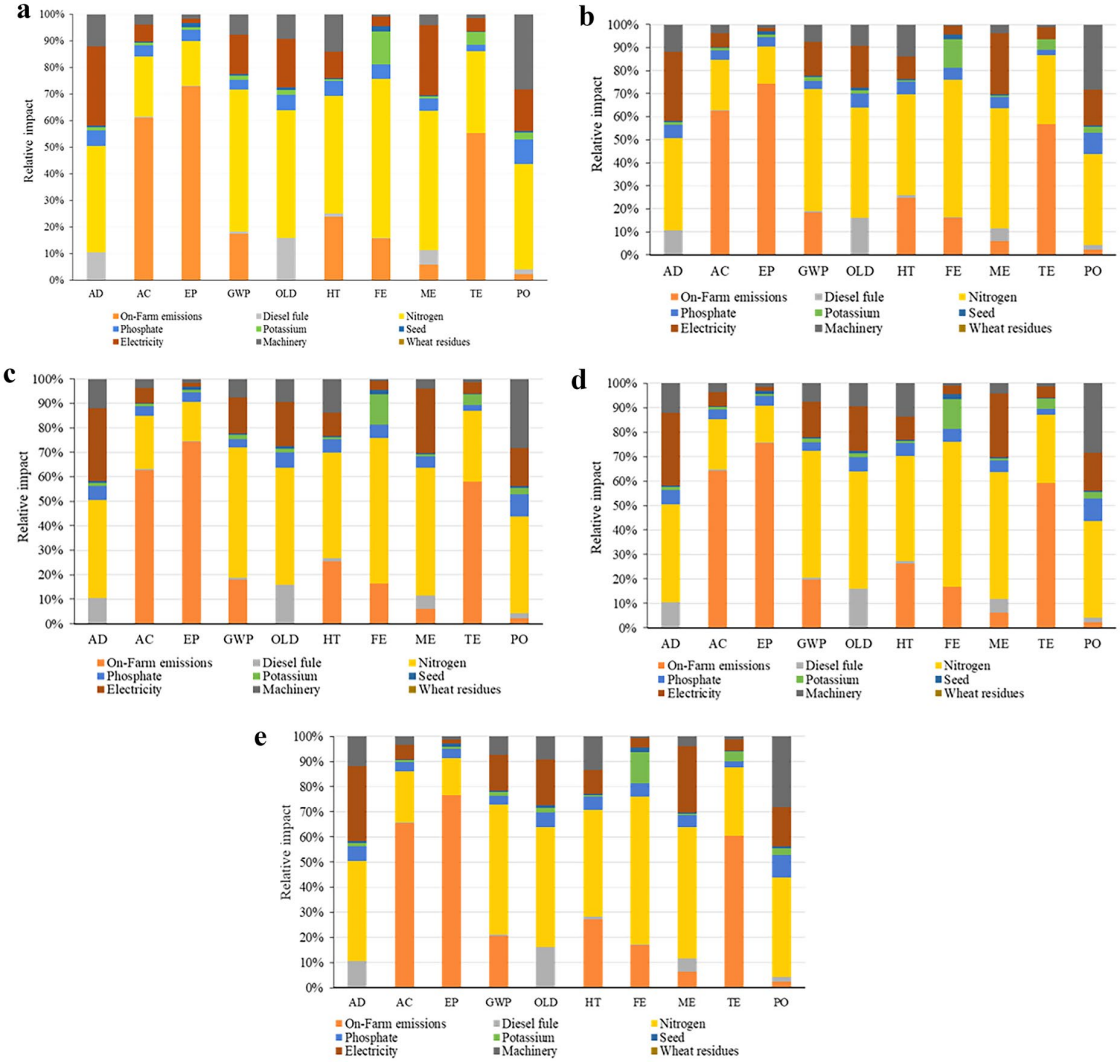
Contribution of **a** 0, **b** 25, **c** 50, **d** 75, and **e** 100% rates of wheat residues in the environmental categories under the CT system in corn silage production

The contribution of different rates of wheat residues on the degradation rate in the CT system is presented in Fig. 2a–e. According to the results, on-farm emissions and nitrogen fertilizers were the two processes with the highest contribution to the degradation rate in terms of environmental parameters at all different rates of wheat residues. The shares of on-farm emissions and nitrogen fertilizer emissions to the different environmental impact categories are shown in Fig. 2a–e.

#### LCA analysis of corn silage in the NT system

The effects of different rates of wheat residues on different environmental impact categories under the NT system in corn silage production are presented in Table 6. The results demonstrated that keeping more wheat residues on fields decreases the overall emissions. Among all the environmental impacts studied, ME and GWP had the highest levels of pollution. The amount of ME at 0, 25, 50, 75, and 100% rates of wheat residues was 17,174 kg, 14,311 kg, 12,710.56 kg, 11,746.96 kg, and 11,442.39 kg 1,4-DB eq., respectively. On the other hand, the values of GWP were 175.6 kg, 147.1 kg, 131.5 kg, 122.3 kg, and 120.2 kg CO_2_ eq., respectively, for the same wheat residue rates. The highest environmental impacts were reported in the scenario without wheat residues present on the field.

**Table 6:** Results for the environmental impacts of the NT system for different rates of wheat residue to produce 1 ton of corn silage

Impact categories	Measurement units	Value				
		0% wheat residue	25%	50%	75%	100%
AD	kg Sb eq	0.58	0.48	0.42	0.39	0.38
AC	kg SO_2_ eq	2.04	1.74	1.58	1.49	1.48
EP	kg ${\text{PO}}_{4}^{3 - }$ eq	0.50	0.43	0.39	0.37	0.37
GWP	kg CO_2_ eq	175.60	147.18	131.55	122.35	120.21
OLD	kg CFC-11 eq	3.94E − 06	3.29E − 06	2.92E − 06	2.69E − 06	2.63E − 06
HT	kg 1,4-DB eq	90.39	75.77	67.70	62.92	50.62
FE	kg 1,4-DB eq	19	15.85	14.09	13.04	12.50
ME	kg 1,4-DB eq	17,174	14,311	12,710.56	11,746.96	11,442.39
TE	kg 1,4-DB eq	4.01	3.39	3.06	2.87	1.60
PO	kg C_2_H_4_ eq	0.03	0.02	0.02	0.02	0.02

*AD* abiotic depletion, *AC* acidification, *EP* eutrophication, *GWP* global warming potential, *OLD* ozone layer depletion, *HT* human toxicity, *FE* freshwater aquatic ecotoxicity, *ME* marine aquatic ecotoxicity, *TE* terrestrial ecotoxicity, *PO* photochemical oxidation

**Fig. 3:**
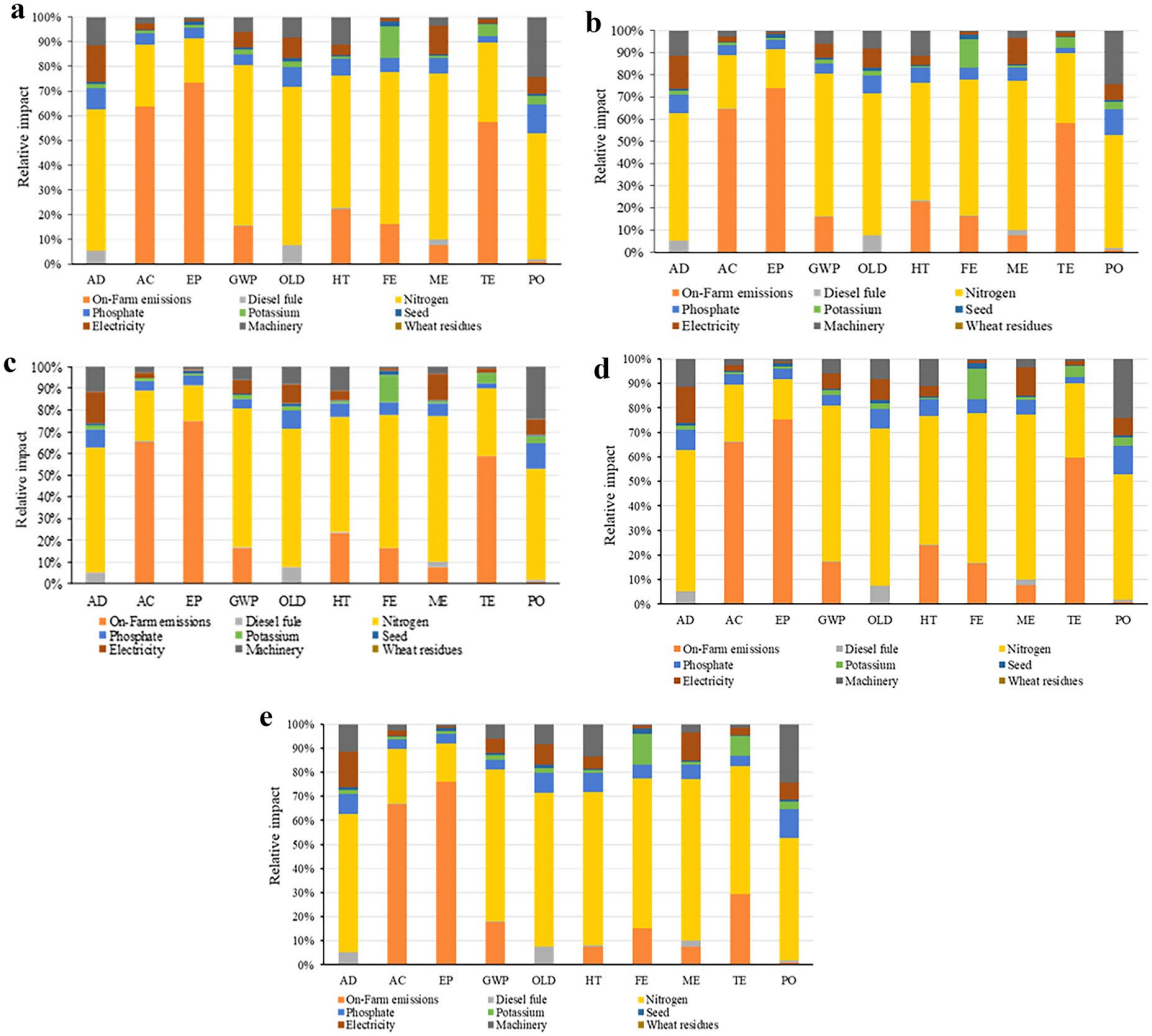
Contribution of **a** 0, **b** 25, **c** 50, **d** 75, and **e** 100% rates of wheat residues in the environmental categories under the NT system in corn silage production

The influence of the different rates of wheat residues on the degradation rate for the NT system is shown in Fig. 3a–e. Our results revealed that on-farm emissions from the farm and nitrogen fertilizers were the two most important factors responsible for degradation related to the environmental impacts, irrespective of wheat residue rates. The contribution of on-farm emissions and nitrogen fertilizer emissions to the different environmental impact categories is presented in Fig. 3a–e.

### Selection of the most sustainable rate of wheat residue

**Fig. 4:**
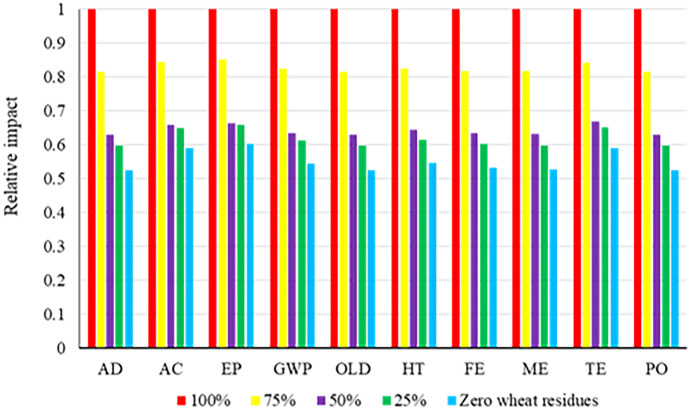
Comparison of total environmental impacts of different application rates of wheat residues used under the CT system

**Fig. 5:**
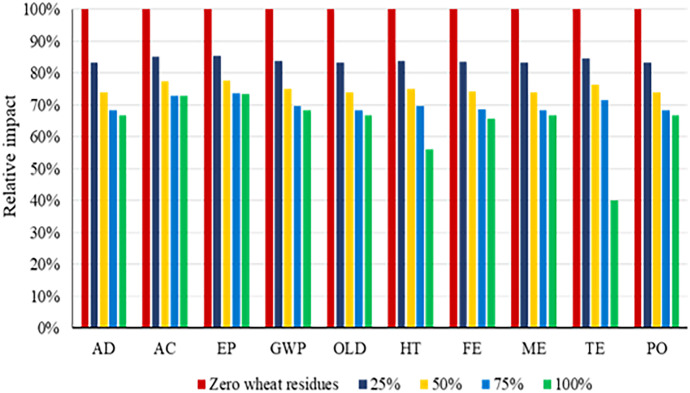
Comparison of total environmental impacts of different application rates of wheat residues used under the NT system

After assessing the damage categories using the LCA method, the best rate of wheat residues for corn silage production in the CT and NT systems was selected. Results show that the 100% rate of wheat residue had the highest level of pollution in the CT system (Fig. 4). The lesser the wheat residues used, the fewer the pollutants released in the CT system. According to the results presented in Fig. 5, the highest levels of contamination in the NT system were reached in the zero-wheat residue used scenario. The higher residue present in the NT system resulted in a lesser number of pollutants.

## Discussion

According to our results, the application of different wheat residue rates in the CT and NT systems showed diverse environmental impacts. On-farm emission and nitrogen fertilizer were reported to have the highest environmental impacts at all rates of wheat residues for corn silage production. Among all the environmental impacts studied, ME and GWP were found to have the highest levels of pollution. Furthermore, the amount of studied environmental impacts (AD, AC, EP, GWP, OLD, HT, FE, ME, TE, and PO) in the CT system was higher than that in the NT system. This is in concordance with several recent research studies conducted on other crops such as corn silage (Fathollahi et al., 2018), wheat (Wang & Dalal, 2015), wheat and maize (Fantin et al., 2017), or orchards such as vineyard grape (Bogunovic et al., 2020; Marques et al., 2020), olive (López-Vicente & Álvarez, 2018; Taguas et al., 2015), and citrus (Niu et al., 2021; Novara et al., 2019).

In addition, in the CT system, the level of environmental impacts raised with an increase in wheat residues was due to rapid decomposition and oxidation processes enhanced by soil disturbance. Returning crop residues on the soil affected soil greenhouse gas emissions and GWP. Crop residues provide carbon and nitrogen for soil-living microorganisms responsible for the production and emission of greenhouse gas (GHG) (Chen et al., 2015; Schmatz et al., 2020; Seiz et al., 2019; Wang et al., 2019). These microorganisms can also affect environmental factors, and their activities strongly influence the emission of GHG and, consequently, GWP (Drury et al., 2020; Schaufler et al., 2010; Wegner et al., 2018).

Using the LCA study, Mdhluli and Harding (2021) reported higher environmental impacts of wheat and maize residues which are in concordance with our findings. Besides, Gabrielle and Gagnaire (2008) reported that environmental emissions are not strongly influenced by the removal of cereal straw in the field. This is in agreement with our findings in no-residue treatment. In contrast to our findings, Parajuli et al. (2017) reported the lowest environmental impact for winter wheat straw. Zhang et al. (2014) investigated the effects of incorporating rapeseed residues into the soil on GWP and noticed that residue-incorporated treatments significantly decreased net GWP by 33–71% over no-residue treatment.

In the NT system, the significance of environmental effects decreased with the increase of wheat residues. Crop residues in the NT system were not incorporated into the soil; therefore, the residues decompose slowly and the amount of emissions released decreases. In addition, the retention of plant residues in this system can improve soil structure and, as a result, reduce soil erosion. Furthermore, residues absorb pollutants and other chemicals used in agriculture and also, consequently, reduce pollutant sources, runoff, and environmental contamination (Mirzaei et al., 2021; Singh & Kaur, 2012; Turmel et al., 2015).

Farm operations are the main cause of the rapid rise of on-farm emissions for both tillage systems. The amount of these emissions in the CT system was higher compared to that in the NT system. The reason is that, in the CT system, many factors, such as machinery, human labor, and fuel, were involved in yield production. On the other side, in the NT system, reduction in consumption of electricity, chemical fertilizers, irrigation water, and less use of farm machinery were important ways of efficient management to mitigate environmental pollutants in the production of silage corn in the studied area.

Previous research had also reported that the use of diesel for agricultural machinery, irrigation, and the use of material inputs are the factors responsible for the environmental impacts of field preparation, cultivation, and harvest (Mdhluli & Harding, 2021). Pishgar-Komleh et al. (2011) studied energy consumption for Iranian corn silage production at different levels of farm areas. They found that total CO_2_ emissions in the production of corn silage amounted to 2.79 million tons, of which 75% is derived from machinery, 21% from diesel, and 4% from chemical fertilizers. In a comparative LCA study of winter wheat and summer maize in China, the results showed that the impacts of AD and EP in maize production systems resulted in the most environmental pollution (Wang et al., 2007). In this study, the main environmental effects in wheat production systems were AD and AC. A study by Wang and Dalal (2015) on wheat production reported that the emissions related to diesel consumption were higher under CT than under NT. Wang et al. (2016) estimated that GHG emissions per ton of dry matter from the processing of corn silage amounted to 155 kg CO_2_ eq.

The majority of Iranian agricultural soils are deficient in terms of organic matter presence and nutrient availability which severely affect crop production (Ayoubi et al., 2018; Mirzaei et al., 2021). Therefore, to achieve the optimal yield, more nitrogen fertilizers are used, resulting in increased GHG emissions and other contaminations (Mirzaei et al., 2022b; Yazdanbakhsh et al., 2020). In addition, the use of poor agronomic practices (i.e., lack of crop rotation, improper methods of fertilizer application and irrigation) reduces nitrogen use efficiency and increases dependence on chemical fertilizers. These are the possible reasons for the increase in nitrogen fertilizer emissions in this study.

The implementation of best management practices such as the inclusion of leguminous plants in crop rotation, retention of post-harvest crop residues, use of cover crops, manure, urease inhibitors, drip irrigation, and fertigation has a significant potential to improve nitrogen use efficiency, increase carbon sequestration, and mitigate GWP and environmental pollution (Cha-un et al., 2017; Guardia et al., 2016; Sanz-Cobena et al., 2016; Tellez-Rio et al., 2015). In line with our results, the results of the LCA study by Iriarte et al. (2010) revealed that the highest environmental impact for both sunflower and rapeseed crops was related to chemical fertilizers.

One of the basic objectives of sustainable agriculture is to ensure that production systems are clean, energy-efficient, and economically profitable. Sustainable agriculture production is facing several challenges including the rapid growth of world population, land degradation, increasing GHG emissions, rapid climate change, limited arable land, loss of biodiversity, single ecosystem service, land use change, increasing industrialization, and low food security (Hanson et al., 2008; Tian et al., 2021). Using new techniques such as conservation agriculture rather than conventional plowing operations, and also driving tractors with maximum efficiency, can significantly reduce fuel consumption and overcome many challenges (Mirzaei et al., 2021, 2022a; Safa et al., 2010). However, the worldwide implementation of the above may be complex due to cultural traditions, lack of knowledge, and difficulties in land preparation in areas with steep slopes or calcareous soils (Bayu, 2020; Rodrigo-Comino et al., 2021; Sastre et al., 2016).

Appropriate management of post-harvest crop residues and tillage improves soil quality and enhances water productivity, crop yield, and soil carbon sequestration while mitigating greenhouse gas emissions and GWP (Brennan et al., 2014; Wegner et al., 2018). Previous studies have shown the effectiveness of conservative tillage as a popular management method due to its ability to protect soil, improve soil fertility, and reduce both fossil fuel consumption and production costs (Margenot et al., 2017; Powlson et al., 2016; Zhang et al., 2018). Improper management of crop residues (e.g., removing and burning residues) can accelerate soil erosion, reduce soil fertility, pollute the environment, contaminate surface as well as underground water, and increase emissions of dust, greenhouse gases, and volatile hydrocarbons (Gupta Choudhury et al., 2014; Mu et al., 2016). However, the use of crop residues in the right way leads to nutrient recycling, soil fertility, soil structure improvement, carbon sequestration, and mitigation of GHG emissions, as well as GW (Meena et al., 2015; Mu et al., 2016).

## Conclusion and final recommendations

To achieve sustainability in the agricultural sector, a full and meaningful analysis of different procedures should be carried out. In Iran, the agricultural sector is suffering from land degradation, climate change, pollution, and many other environmental issues. Annually, a massive amount of crop residues is produced, but these residues are burned or used for other purposes, where one of the most widely performed cropping practices in Iran is the wheat–corn rotation system. Thus, we investigate the environmental impacts of corn silage production in terms of varying wheat residue rates under conventional and no-tillage systems using the LCA approach. The output of this research can be summarized as follows:

1. The inputs for the CT system are higher than those for the NT system. The highest amount of CO_2_ released from diesel is associated with the CT system due to the high consumption of diesel for land preparation, cultivation, and harvesting operations.
2. In both tillage systems, on-farm emissions and nitrogen fertilizers were the two important factors with the highest contribution to degradation in terms of environmental parameters at all different rates of wheat residues.
3. ME and GWP had the highest levels of pollution among all the environmental impacts studied.
4. In the NT system, keeping more wheat residues on fields decreases the overall emissions.
5. This research reported a contradictory impact of wheat residues. Hence, the 100% rate of wheat residues has the highest levels of contamination in CT. In contrast, the highest levels of contamination in the NT system were reached in the zero-wheat residue scenario.

All in all, to improve the environmental performance of the biomass energy sector, improvements in agricultural practices should be implemented, including further research on fertilization, water use, agricultural practices, land transformation, biomass conversion technologies, and transportation. However, the output of this research will support decision-makers and researchers in adopting the best agricultural practices to minimize land degradation and GHG emissions.

## Supplementary Information

Below is the link to the electronic supplementary material.

Supplementary file1 (DOCX 75 KB)

## Data Availability

The data that support the findings of this study are available from the corresponding author, (A. C. C.), upon reasonable request.

## Abbreviations

AC: Acidification
AD: Abiotic depletion
CC: Complete coverage
CT: Conventional tillage
DB: Dichlorobenzene
EP: Eutrophication
FE: Freshwater aquatic ecotoxicity
FU: Functional unit
GHG: Greenhouse gas
GW: Global warming
GWP: Global warming potential
HC: Hydrocarbons
HT: Human toxicity
ME: Marine aquatic ecotoxicity
NMVOC: Non-methane volatile organic compound
OLD: Ozone layer depletion
PAH: Polycyclic hydrocarbons
PC: Partial coverage
PM: Particulate matter
PO: Photochemical oxidation
TE: Terrestrial ecotoxicity
