# Supplementary material for: Environmental impacts of corn silage production: influence of wheat residues under contrasting tillage management types
Source: Environ Monit Assess. 2022 Dec 2;195(1):171. doi: 10.1007/s10661-022-10675-8 (PMC9718881; doi:10.1007/s10661-022-10675-8)
Supplement: Supplementary file 1 — Supplementary file1 (DOCX 75 KB) [file 10661_2022_10675_MOESM1_ESM.docx]

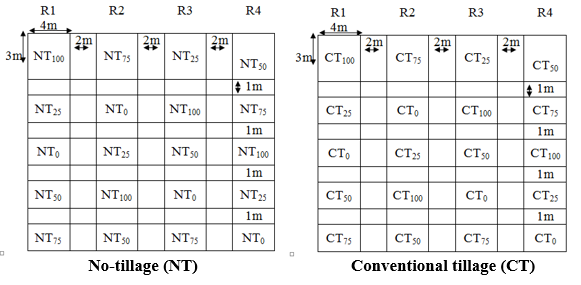


Fig S1. View of the experimental design in tillage systems

| **Table S1**  A literature review of the previous studies undertaken on LCA in agricultural crop production. | | | | |
| --- | --- | --- | --- | --- |
| Surveyed research | Surveyed location | Crop | LCA | Hotspot |
| Pishgar Komleh et al. [2011] | Iran (Tehran) | Silage corn | GHG | Machinery |
| Boulard et al. [2011] | France | Tomato | PC | Biocides |
| Page et al. [2012] | Australia | Tomato | GHG | Transportation |
| Thanawong et al. [2014] | Thailand | Paddy rice | PC | Nitrates, phosphates, and biocides |
| Syp et al.[2015] | Poland | Wheat | GHG | Nitrogen fertilizer and diesel fuel |
| Bacenetti et al. [2015] | Italy | Cereal silage | CC | Diesel fuel |
| Noya et al. [2015] | Italy | Feed cereals | CC | Chemical fertilizers, machinery, and diesel fuel |
| Masuda [2016] | Japan | Wheat | CC | Nitrogen fertilizer |
| Ullah et al. [2016] | Pakistan | Cotton | CC | Biocides and fertilizers |
| Nabavi-Pelesaraei et al. [2017b] | Iran (Guilan) | Paddy | CC | Chemical fertilizers |
| Khanali et al. [2017] | Iran (Khorasan) | Saffron | CC | Chemical fertilizers |
| Houshyar and Grundmann [2017] | Iran (Fars) | Wheat | CC | Chemical fertilizers and diesel fuel |
| Mousavi-Avval et al. [2017a] | Iran (Mazandaran) | rapeseed | CC | Chemical fertilizers |
| Fathollahi et al. [2018] | Iran (Karaj) | Silage | CC | Chemical fertilizers |
| Liang et al. [2019] | China | Winter wheat-summer maize rotation system | CC | Chemical fertilizers |
| Kaab et al. [2019a] | Iran (Ahwaz) | Sugarcane | CC | Electricity, machinery, and biocides |
| Grados and Schrevens [2019] | Peru | Potato | PC | Machinery |

**Table S2.** Soil properties of 0 ‒ 10 and 10 ‒ 20 cm soil depths in the fields in 2019.

| Soil properties | Conventional Tillage (CT) | |  | No-Tillage (NT) | |
| --- | --- | --- | --- | --- | --- |
|  | 0 ‒ 10 cm | 10 ‒ 20 cm |  | 0 ‒ 10 cm | 10 ‒ 20 cm |
| pH | *7.8 ± 0.1 | 7.7 ± 0.1 |  | 7.8 ± 0.0 | 7.6 ± 0.1 |
| EC (ds m^-1^) | 0.9 ± 0.0 | 0.7 ± 0.2 |  | 1± 0.0 | 0.7 ± 0.2 |
| OC (mg/g) | 0.9 ± 0.1 | 0.8 ± 0.2 |  | 1.2 ± 0.0 | 1.01 ± 0.1 |
| TN (mg/g) | 0.09 ± 0.01 | 0.07 ± 0.01 |  | 0.1 ± 0.0 | 0.08 ± 0.0 |
| Avail K (mg kg^-1^) | 167 ± 4 | 134 ± 3 |  | 279 ± 5.2 | 237.4 ± 6.0 |
| Avail P (mg kg^-1^) | 9 ± 0.2 | 9.5 ± 0.6 |  | 15 ± 1.5 | 15.3 ± 0.7 |
| Bulk density (g cm^−3^) | 1.52 | 1.56 |  | 1.32 | 1.38 |
| Total Porosity (%) | 54 ± 2 | 52± 1.1 |  | 48 ± 1.9 | 45 ± 1 |
| Sand (%) | 57 ± 1 | 53± 1 |  | 28 ± 0.3 | 27 ± 0.7 |
| Silt (%) | 25 ± 1 | 28± 1 |  | 42 ± 0.2 | 42 ± 0.6 |
| Clay (%)  Soil texture | 18 ± 1  Sandy Loam | 19 ± 2  Sandy Loam |  | 30 ± 0.4  Clay Loam | 31 ± 0.7  Clay Loam |

EC: electrical conductivity, OC: organic carbon, TN: total nitrogen, Avail K: available potassium, Avail P: available phosphorus.

*Values are Mean± Standard Deviation (n=3).

**Table S3.** Chemical composition of wheat residue.

| Plant residue | N | P | K | C | C/N |
| --- | --- | --- | --- | --- | --- |
|  | % | | | | - |
| Wheat | *0.84±0.12 | 0.09±0.01 | 1.75±0.07 | 55.4±1.80 | 66±2.56 |

*Values are Mean± standard deviation (n=3).

**Table S4.** Monthly precipitation (mm) and mean air temperature (˚C) during the period of the experiment.

| Corn growing season (2018) | Mean air temperature (˚C) | Monthly precipitation (mm) |
| --- | --- | --- |
| July | 32 | None |
| August | 29 | None |
| September | 24 | None |
| October | 17 | None |
